# The truth revisited: Bayesian analysis of individual differences in the truth effect

**DOI:** 10.3758/s13423-020-01814-8

**Published:** 2020-10-26

**Authors:** Martin Schnuerch, Lena Nadarevic, Jeffrey N. Rouder

**Affiliations:** 1 Department of Psychology, School of Social Sciences, University of Mannheim, 68131 Mannheim, Germany; 2 University of California, Irvine, CA USA

**Keywords:** Individual differences, Qualitative differences, Truth effect, Hierarchical models, Bayesian model comparison

## Abstract

The repetition-induced truth effect refers to a phenomenon where people rate repeated statements as more likely true than novel statements. In this paper, we document *qualitative* individual differences in the effect. While the overwhelming majority of participants display the usual *positive* truth effect, a minority are the opposite—they reliably discount the validity of repeated statements, what we refer to as *negative* truth effect. We examine eight truth-effect data sets where individual-level data are curated. These sets are composed of 1105 individuals performing 38,904 judgments. Through Bayes factor model comparison, we show that reliable negative truth effects occur in five of the eight data sets. The negative truth effect is informative because it seems unreasonable that the mechanisms mediating the positive truth effect are the same that lead to a discounting of repeated statements’ validity. Moreover, the presence of qualitative differences motivates a different type of analysis of individual differences based on ordinal (i.e., Which sign does the effect have?) rather than metric measures. To our knowledge, this paper reports the first such reliable qualitative differences in a cognitive task.

In the usual course of experimental psychology, we often understand phenomena by computing the mean effect. This mean effect may be used to compute effect sizes or statistical tests, and the resulting inferences are about the mean level in the population. In our view, this focus on the mean makes sense when all people experience a phenomenon in a qualitatively similar way. For example, suppose we ask people to identify a briefly presented and subsequently masked letter. In this case, increasing the stimulus duration of the letter should affect every individual in the same direction, namely that longer durations correspond to better performance. It seems implausible in fact for any person’s true performance to decrease with increasing stimulus duration, and it is in this sense where we can be almost sure that a phenomenon affects people in a qualitatively similar manner, that recourse to the mean seems judicious.

What happens if a treatment affects different people differently? A good example might be the effect of aspirin. For most people, the drug aspirin safely relieves pain. Yet, a minority of the population are allergic to aspirin, and for these people the allergic reaction may be serious. In this case, questions about the mean response seem unimportant. Instead, the important questions are what proportion of the population is allergic and what are the separate mechanisms of pain relief and allergic reactions.

The question of whether the overall mean is useful hinges on whether an effect is qualitatively consistent across individuals. In the first example, it seems implausible that increasing the stimulus duration of a briefly flashed and subsequently masked object could decrease identification for anyone. For this example, the average performance gain as a function of stimulus duration seems a reasonable target for inquiry. For the aspirin example, however, average gain in pain relief seems far less helpful.

The key methodological question is how to tell if an effect is qualitatively consistent across a set of participants. Progress on this question has been made by Haaf and Rouder and colleagues (Thiele et al.,, 2017; Haaf & Rouder, 2017, 2019). We will review their approach subsequently, but for now, their research has yielded a startling finding. When it comes to performance tasks, it seems that people don’t qualitatively differ. For example, nobody truly responds quicker to incongruent items in Stroop, Simon, or flanker tasks. Indeed, we are previously unaware of any case where substantial qualitative differences appear in a performance task. And this paper is the first we are aware of that shows such differences.

In this paper, we explore individual differences in a truth-judgment task. More precisely, the target of inquiry is a popular psychological effect, the repetition-induced *truth effect*. In a typical truth-effect task, participants rate how likely it is that a particular statement is true. The critical manipulation is repeating some statements, and these repeated statements are more likely rated as true than novel ones. The real-world impact of the truth effect is obvious: If a lie is repeated, it is more likely to be believed.

The question we ask is whether all people are susceptible to what we call a *positive* truth effect where repeated statements are judged as more valid than novel ones. The alternative is that some people have a true *negative* truth effect where they tend to discount the validity of repeated statements.

We find that researchers often stake out positions about individual differences *a priori*, and believe them with a surprising degree of confidence. One position we encounter is what we call the *arbitrary diversity hypothesis* (Rouder & Haaf, 2020). Accordingly, the human condition is so diverse that there must be people who deviate in all behaviors. Indeed, while it may be plausible to ascribe stringent constraint in low-level cognitive behaviors such as perception and attention, it seems far less plausible that such constraint holds in high-level tasks like judging the truth of a statement. Different people almost surely use different processes, heuristics, anchors, and values in making such judgments. Despite the intuitive appeal of the arbitrary diversity hypothesis, we think it is a mistake to put too much stock in it *a priori*. The reasons are because (a) it is an empirical question, and (b) it precludes notions of lawfulness and constraint. Moreover, even if we noticed diversity in behavior, would we not be obligated to try to find deeper invariances that are preserved? For example, rational choice theory does this through expectation of subjective utility. We might disagree on utilities, but we all maximize our own. The arbitrary diversity hypothesis, if taken as the last word, is throwing out the baby with the bath water. We would prefer that researchers test this hypothesis carefully in data, and that is our goal with the truth effect.

We analyze data from eight previous experiments spanning 1105 participants and 38,904 trials. We show to our surprise that in all the data sets where variation is detectable, there are some people who have a reliably negative truth effect. Most people show a positive truth effect, but a small minority truly discount the validity of repeated information.

## The truth effect

Repeatedly encountering a piece of information is likely to increase the subjective belief in its validity. This phenomenon has been known—and used—for thousands of years. Around 150 BC, the Roman politician Cato reportedly concluded each of his speeches with the same sentence: “Carthago delenda est” (*Carthage must be destroyed*). It seems that he succeeded in convincing the Roman Senate to approve of his proposition: In 146 BC, Carthage was destroyed. Cato’s strategy might have resonated well with French military leader Napoleon Bonaparte, who conquered large parts of Europe in the early 19th century. According to Bonaparte, “there is only one figure in rhetoric of serious importance, namely, repetition” (Le Bon, 1895, p. 125). Roughly 100 years later, Nazi German Minister of Propaganda Joseph Goebbels made use of this rhetorical device; “Repeat a lie often enough and it becomes the truth”, is typically attributed to Goebbels (Stafford, 2016).

The effect of repetition on the perception of a proposition’s truth is a well-documented phenomenon in experimental psychology. In a seminal study, Hasher et al., (1977) asked participants to provide truth ratings for trivia statements in successive sessions. Critically, some of the statements were repeated across sessions while others were novel. The authors found validity ratings for repeated statements to increase, independent of the statements’ actual validity, while ratings for novel statements did not change.

Over the past 40 years, the truth effect has been replicated numerous times (Dechêne et al., 2010; Unkelbach et al., 2019). It has been shown to be robust across different experimental designs, material, and instructions. Even explicit warnings about the effect do not eliminate it, but only reduce it (Nadarevic and Aßfalg, 2017). The consequences of such a robust cognitive bias are evident: If used strategically, repeated dissemination increases belief even in false information (Unkelbach et al., 2019; Lazer et al., 2018; Pennycook et al., 2018).

Meta-analytic results indicate that the truth effect is stable across studies, and is medium in size (Cohen’s *d* ≈ 0.50; Dechêne et al., 2010). Consequently, the truth effect has been well established for the average of individuals. In contrast, we are aware of only a small number of published studies on individual differences (Arkes et al., 1991; Boehm, 1994; Brashier et al., 2017; De Keersmaecker et al., 2020; Newman et al., 2020; Parks & Toth, 2006). All of these studies assessed the covariation of individual truth effects and certain person-specific variables (e.g., age, need for cognition, and cognitive style). Yet, correlational analyses do not address the main question here, namely: Are individual differences only quantitative, or qualitative?

*Quantitative* differences occur if all participants provide somewhat higher truth ratings for repeated than for novel statements. We might reasonably assume a common process underlying the effects in this case, and the mean might even be an adequate representation for understanding this process. The assumption is less reasonable, however, if differences are *qualitative*, that is, if some participants were to depreciate the validity of repeated statements. Indeed, qualitative individual differences are precedented in the domain of truth judgments (i.e., belief polarization; Cook & Lewandowsky, 2016). Is it still the same process that leads some people to increase their belief in repeated statements and others to decrease it? If qualitative differences can be shown, this has theoretical implications. A theory of the truth effect would have to account for both the increase and the decrease in beliefs due to repetition. Therefore, to gain constraint on theory, a fundamental question in the analysis of the repetition-induced truth effect is what Haaf and Rouder (2017) coined the “Does everybody?” question: Does everybody show a positive truth effect?

This fundamental question comes with a methodological challenge: Even if we observe a negative truth effect for some individuals, this observation might reflect sampling noise rather than true qualitative differences. How do we assess whether people truly differ and, if so, whether these differences are qualitative? To answer these questions, we follow the strategy proposed by Haaf and Rouder (2017, 2019). We develop a set of hierarchical Bayesian models that represent different structures of individual differences. By means of model comparison, we then directly assess the evidence from data about the nature of individual differences.

In the following, we provide a brief description of the eight data sets that we reanalyze in this study. We then develop the statistical models of individual-differences structures and outline the procedure to quantify evidence for these models. With model comparison, we find a surprising result: Across many of the data sets, there is a small proportion of individuals that show a negative truth effect.

## Data sets

We reanalyzed eight data sets from previous truth-effect experiments, all of which are publicly available from the Open Science Framework (OSF).^1^ Six of the sets have been published in peer-reviewed articles; two have been published only on OSF. Detailed information about the data and the experiments can be obtained from Appendix A.

All data sets are based on a common experimental design with three phases. Phase 1 is the *exposure phase*: Participants see a number of trivia statements and, typically, assign each statement to a semantic domain (e.g., biology, geography, sports) or rate each for interest. Phase 2 is a retention interval, in which participants may perform an unrelated task. This phase can range from a few minutes to several days. Phase 3 is the critical *judgment phase* where participants rate the validity of statements. Ratings are typically given on a Likert scale, for example, from 1 (“definitely false”) to 6 (“definitely true”). Critically, half of the statements have been presented during the exposure phase and half are new. The truth effect is measured as the difference between mean truth ratings for repeated and for new statements, *M*_*rep*_ − *M*_*new*_.

For an overview of sample characteristics and results of the eight data sets, see Table 1. For the sake of comparison, we rescaled truth ratings to range from − 1 (“definitely false”) to 1 (“definitely true”). As a consequence, the truth effect can range from − 2 to 2. If all repeated statements received truth ratings of 1 while all new were rated as − 1, the resulting truth effect would be 2. An effect of − 2, in contrast, would indicate a perfect reversal of the truth effect. Zero represents the absence of any effect.

**Table 1:** Summary of the data sets

Set	Source	*N*	*I*	Mean effect*	*t* Test	Cohen’s *d*
1	Nadarevic et al., (2012)	267	20	0.20 (0.34)	*t*(266) = 9.47	0.58
2	Nadarevic and Rinnewitz (2011)	139	20	0.28 (0.36)	*t*(138) = 9.16	0.78
3	Nadarevic and Aßfalg (2017), Exp. 1	33	88	0.11 (0.10)	*t*(32) = 6.54	1.14
4	Nadarevic and Aßfalg (2017), Exp. 2	98	80	0.20 (0.23)	*t*(97) = 8.61	0.87
5	Nadarevic and Erdfelder (2014), Exp. 1	85	88	0.05 (0.10)	*t*(84) = 5.09	0.55
6	Nadarevic and Erdfelder (2014), Exp. 2	35	88	0.07 (0.17)	*t*(34) = 2.51	0.42
7	Brashier et al., (2020), Exp. 1	52	120	0.09 (0.12)	*t*(51) = 5.52	0.77
8	Pennycook et al., (2018), Exp. 1	396	20	0.14 (0.27)	*t*(395) = 10.08	0.51

*Note.* *N* = Number of participants; *I* = Number of statements rated per participant; *t* values and degrees of freedom are based on paired *t* tests. *Standard deviation is given in parentheses.

**Fig. 1:**
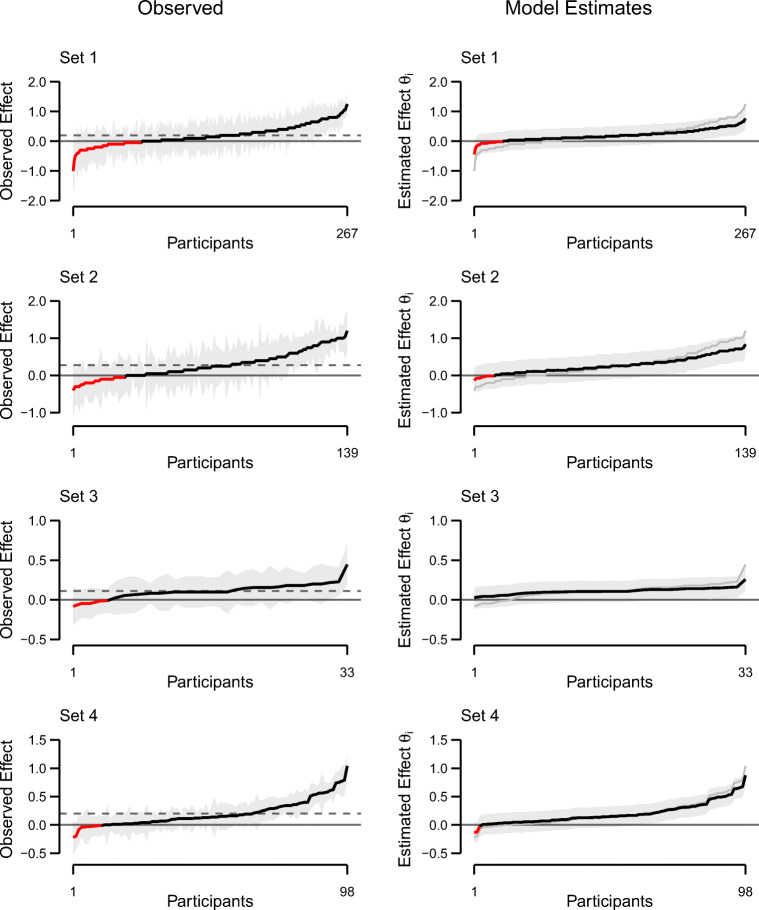
Observed (*left column*) and estimated (*right column*) individual truth effects for data sets 1–4, ordered by observed effect size. On the left side, the *shaded area* denotes individual 95% confidence intervals. The *dashed line* represents average observed effects. On the right side, the *shaded area* denotes the 95% credible interval. The *grey line* represents observed truth effects. Negative observed and estimated effects (i.e., higher truth ratings for novel than for repeated statements) are denoted by *red color* on both sides

**Fig. 2:**
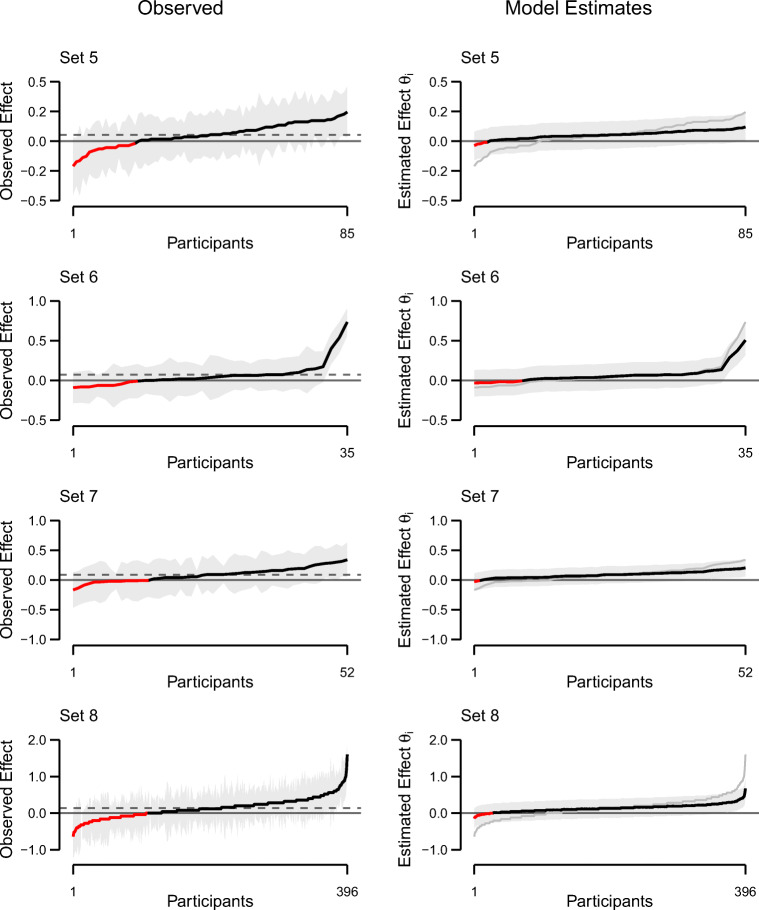
Observed (*left column*) and estimated (*right column*) individual truth effects for data sets 5–8, ordered by observed effect size. On the left side, the *shaded area* denotes individual 95% confidence intervals. The *dashed line* represents average observed effects. On the right side, the *shaded area* denotes the 95% credible interval. The *grey line* represents observed truth effects. Negative observed and estimated effects (i.e., higher truth ratings for novel than for repeated statements) are denoted by *red color* on both sides

Figures 1 and 2 (left columns) show the individual truth effects in all eight data sets (black line). Individuals are sorted by the size of their effect, going from the most negative to the most positive. The red line indicates if the individual effect is below 0, that is, negative observed truth effects. The grey-shaded area surrounding the line denotes 95% confidence intervals. The average effect across all people is given by the dashed horizontal line. In all data sets, we observe considerable differences between individual participants.

## Statistical models

The main substantive question is whether individual differences are quantitative or qualitative. Our strategy in answering this question is to implement each of these positions in statistical models, and then compare the models in light of data with Bayes factors. The specific models come from Haaf and Rouder (2017), and, consequently, we provide only a brief overview here. Let *Y*_*ijk*_ denote the truth judgment of the *i* th person (*i* = 1, … , *I*) for the *j* th statement (*j* = 1, … , *J*) in condition *k* (*k* = 1, 2 for new versus repeated, respectively). Note that not every statement *j* is necessarily seen by each participant *i*. Consequently, the data sets do not contain *Y*_*ijk*_ for every possible combination of *i*, *j*, and *k*. This fact presents no problem in analysis.

We specify the following linear model on the dependent variable:

1 $$ Y_{ijk} \overset{\text{ind}}{\sim} \text{Normal} (\mu + \alpha_{i} + t_{j} \beta + x_{k} \theta_{i},\ \sigma^{2}). $$

In this model, *μ* denotes the grand mean intercept and *α*_*i*_ is a person-specific deviation from this grand mean. The term *t*_*j*_ codes the truth status of the *j* th statement, which can either be 0 if it is false, or 1 if it is true. Hence, *β* denotes the effect of a statement’s factual truth on the judgment. The term *x*_*k*_ codes the repetition condition, which can either be 0 if the statement is new or 1 if it is repeated. Consequently, *θ*_*i*_ denotes the *i* th individual’s truth effect, and this parameter is the main target of inquiry. The last term, *σ*^2^, denotes the sampling variance of observed values. The main theoretical positions about individual differences motivate the following four models on *θ*_*i*_:

### Unconstrained model

The unconstrained model, ${\mathscr{M}}_{u}$, does not impose any constraints on the individual effects. It may be used to capture qualitative individual differences:

2 $$ \mathcal{M}_{u}\text{: }\theta_{i} \overset{\text{iid}}{\sim} \text{Normal}(\nu,\ \delta^{2}). $$

In this model, *ν* and *δ*^2^ denote the mean and variance of individual effects. These group-level parameters are estimated from the data.

### Positive-effects model

The positive-effects model, ${\mathscr{M}}_{+}$, is less flexible. It only allows for positive individual effects:

3 $$ \mathcal{M}_{+}\text{: }\theta_{i} \overset{\text{iid}}{\sim} \text{Normal}_{+}(\nu,\ \delta^{2}). $$

The distribution denoted by Normal_+_ is a truncated normal with a lower bound at zero.^2^ Thus, the model naturally incorporates the constraint that individuals may differ but they are all in the same predicted direction. Substantively, this model implies that differences are quantitative, but not qualitative.

### Common-effect model

The critical specification in the common-effect model, ${\mathscr{M}}_{1}$, is that all individuals share one common effect:

4 $$ \mathcal{M}_{1}\text{: }\theta_{i} = \nu. $$

Accordingly, there are no true individual differences in the truth effect. Any observed variation would thus be due to sampling noise.

### Null Model

The final model is a null model, ${\mathscr{M}}_{0}$, where there is no truth effect at all:

5 $$ \mathcal{M}_{0}\text{: }\theta_{i} = 0. $$

Accordingly, any observed effects of statement repetition are due to sampling noise.

**Fig. 3:**
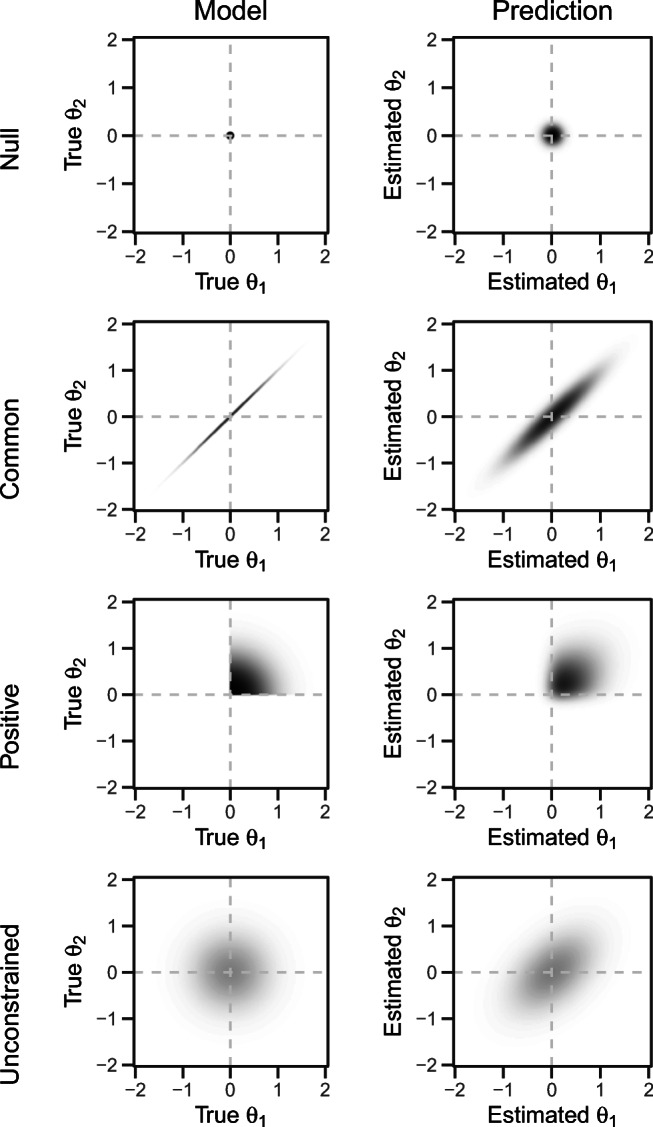
Illustration of the four models (*left column*) on *θ*_*i*_ and corresponding predictions (*right column*) for two hypothetical participants

Figure 3 (left column) illustrates the four models. On the *x*-axis, the true effect of a hypothetical participant is shown, *θ*_1_. On the *y*-axis, the true effect of a second participant is shown, *θ*_2_. The null model specifies that both effects are 0, thus, the model is represented by a point at the origin. The common-effect model does not restrict true effects to one value, but specifies that all individual effects are identical. This is represented by the diagonal line. No equality constraints are imposed in the positive-effects model, but all true individual effects are defined as larger than zero. Accordingly, *θ*_1_ and *θ*_2_ are free to vary in the upper-right quadrant. Finally, the unconstrained model puts no restrictions on individual effects; the model for the two hypothetical participants is represented by a bivariate normal centered at the origin.

### Prior specification

We implement these models in the Bayesian framework, and, as such, priors are needed on parameters. For some parameters, those that are common in all four models, the priors may be set without undue influence on the posteriors or model comparison statistics. These specifications are provided in Appendix B. For other parameters, however, those that vary across models (*θ*_*i*_, *ν*, *δ*^2^), prior settings are important and are discussed here.

We follow the common *g*-prior specification approach (Zellner, 1986), which is based on placing priors on effect sizes. The setup is described in detail in Haaf and Rouder (2017) and Rouder et al., (2012). Let *g*_*θ*_ be a signal-to-noise ratio defined as *g*_*θ*_ = *δ*^2^/*σ*^2^. This is an effect-size description of *θ*; it describes how much true variability there is across people relative to the variability in observations. With this parameter, we may write $\theta _{i} \sim \text {Normal}(\nu ,g_{\theta }\sigma ^{2})$. Priors are needed on *ν* and *g*_*θ*_. The prior on *ν* is also scaled to the variability in observations: $\nu \sim \text {Normal}(0,g_{\nu }\sigma ^{2})$, and there is a new parameter *g*_*ν*_. Priors on these *g* parameters are Inverse-*χ*^2^ distributions with one degree of freedom and a scale parameter *r*^2^:

6 $$ \begin{array}{ll} g_{\nu} & \sim \text{Inverse-}\chi^{2}(r_{\nu}^{2}),\\ g_{\theta} & \sim \text{Inverse-}\chi^{2}(r_{\theta}^{2}). \end{array} $$

**Fig. 4:**
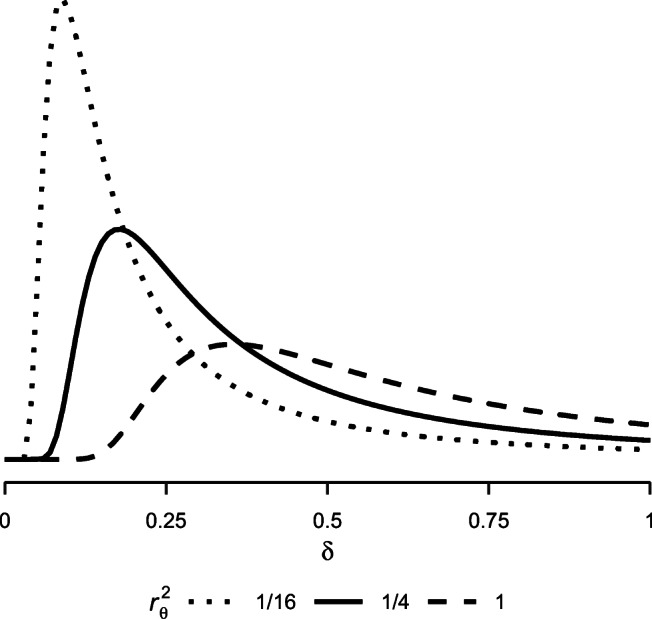
Prior distributions on *δ*, the variability of *θ*_*i*_, for different scale settings. A trial-by-trial variation of *σ* = 0.50 is assumed

Researchers need to set the scales of these priors before the analysis. We advocate that doing so should rely on substantive considerations rather than statistical arguments. Here is our line of thought: In our experience, on a standardized scale of − 1 to 1, truth judgments’ trial-by-trial variability covers about a quarter of the scale, that is, *σ* = 0.50. As a reference, on a scale from 1 to 6, this corresponds to a standard deviation of *σ* = 1.50. When specifying *r*, it is helpful to consider that it represents an expectation about the variability of the parameter relative to *σ*. For example, a value of *r*_*θ*_ = 1 encodes the belief that the variability of person-specific truth effects (i.e., *δ*) is comparable to the trial-by-trial variability. Likewise, *r*_*θ*_ = 1/2 or *r*_*θ*_ = 2 represent the expectation that *δ* scales about half or about twice as large as *σ*, respectively. Figure 4 illustrates the effect of different choices of *r*_*θ*_. It shows the resulting prior distributions on the variability of *θ*_*i*_ conditional on a trial-by-trial variability of *σ* = 0.50.

Based on this information, how do we choose the scale parameters? The average truth effect is of medium size, Cohen’s *d* = 0.50 (Dechêne et al., 2010). Assuming *σ* = 0.50, the expected observed effect on a rating scale of − 1 to 1 is thus 0.25. We find it reasonable to expect that the variability of this effect is of a comparable magnitude. Therefore, we define the scales of our *g* priors on *θ*_*i*_ and *ν* such that they place most weight on values around half the trial-by-trial variability, that is, $r_{\nu }^{2} = r_{\theta }^{2} = (1/2)^{2}$.

### Model comparison

The models may be used to answer the main substantive question about qualitative individual differences. One approach is simply to estimate individual effects (*θ*_*i*_) in the full model. Yet, we think this approach is ultimately unhelpful. The problem is that whether there are qualitative differences between individuals is a global property rather than an individual one. One may know that someone must be negative without the ability to identify who. Consequently, individual estimates are not helpful in themselves. And population-level mean effects are not helpful either as they do not address the distinction between qualitative and quantitative individual differences. Hence, the key to assessment here may be made with model comparison and not with estimation.

A leading approach to model comparison in Bayesian analysis is the *Bayes factor* (Jeffreys, 1935, 1961). Bayes factors measure the relative strength of evidence for models by comparing how well these models predict the data (Rouder and Morey, 2017; Kass & Raftery, 1995). Figure 3 (right column) illustrates the predictions that the different models make for observed data of the two hypothetical participants. These predictions are noisy versions of the structure on true values. The more flexible a model is, that is, the fewer restrictions it imposes on the structure of individual differences, the more diffuse are its predictions. Hence, models are penalized for flexibility.

Analysis for the null model, the common-effect model, and the unconstrained model have been developed by Rouder et al., (2012). Their approach is implemented in the *BayesFactor* package (Morey and Rouder, 2015) in R (R Core Team, 2019), which allows for fast and accurate calculation of Bayes factors for three of the four models. This development, however, does not apply to the positive-effects model. Therefore, we calculated the Bayes factors between the positive-effects and the unconstrained model using the *encompassing prior* method proposed by Klugkist and colleagues (Klugkist & Hoijtink, 2007; Klugkist et al., 2005). The combination of these two approaches is straightforward (e.g., Haaf & Rouder, 2017,2019).

## Evidence for qualitative differences

### Model convergence

Posterior distributions for all parameters in the unconstrained model are obtained by Markov chain Monte Carlo (MCMC) sampling within the BayesFactor package. We checked model convergence by inspecting MCMC chains and computing autocorrelations for critical parameters (i.e., *ν*, *θ*, *g*_*ν*_, and *g*_*θ*_). As in previous applications, the models converged fast and the chains mixed well. The autocorrelations for even the slowest converging parameters were inconsequential compared to the large number of posterior samples (10,000).

### Estimation

Individual truth effect estimates from the unconstrained model for all eight data sets are shown in the right columns of Figs. 1 and 2. The black line denotes the posterior means of *θ*_*i*_ for each participant. The grey band around this line is the 95% credible interval, that is, an interval that contains 95% of the posterior samples. The grey line represents observed individual truth effects and the ordering is obtained from these observed values (see left columns).

The first aspect to note is the effect of the hierarchical model specification on individual estimates. The effect is called shrinkage: Individual estimates inform each other and thus, outliers are pulled (*shrunk*) toward the mean. This shrinkage is clearly visible in the estimates; there is less variability than in the observed effects. Note that the credible intervals are much smoother than the individual confidence intervals of observed effects (left column), reflecting regularization from the homogeneous variance specification. The second aspect to note is that even with the shrunk estimates, considerable true individual differences remain. And the third aspect, perhaps the most consequential, is that some of these shrunk estimates are negative.

### Model comparison

Table 2 summarizes the results of the Bayes factor model comparison for the eight data sets. In each column, an asterisk marks the *preferred* model, the one for which the data provided the most evidence. The other cells in each column show the Bayes factors between the remaining models and this preferred model. Because these remaining models are less preferred, the Bayes factors are always less than one, mostly by many orders of magnitude.

**Table 2:** Bayes factor model comparison

	Data set							
Model	1	2	3	4	5	6	7	8
$\mathcal M_{0}$	3.4e-58	5.2e-57	4.6e-08	1.8e-124	9.2e-05	2.9e-07	4.3e-07	5.4e-41
$\mathcal M_{1}$	1.4e-20	1.1e-16	*	1.1e-49	*	6.1e-05	*	3.1e-09
$\mathcal M_{+}$	1.2e-03	1.1e-03	2.4e-03	1.0e-03	1.6e-07	9.3e-04	2.3e-05	1.2e-03
$\mathcal M_{u}$	*	*	2.2e-03	*	1.5e-04	*	2.0e-03	*

*Note.* The preferred model for each data set is indicated by an asterisk. Remaining cells contain Bayes factors for each model against the preferred model. $\mathcal M_{0}$ = null model; $\mathcal M_{1}$ = common-effect model; $\mathcal M_{+}$ = positive-effects model; $\mathcal M_{u}$ = unconstrained model.

In five data sets, we find strong evidence for qualitative differences: In sets 1, 2, 4, 6, and 8, the Bayes factors in favor of the unconstrained model ${\mathscr{M}}_{u}$ are at least 1000-to-1 over the next leading competitor. As ${\mathscr{M}}_{u}$ is the only model that allows for qualitative individual differences, the Bayes factors provide compelling evidence for them. In these data sets, there must be some individuals with a true negative truth effect.

In the remaining three data sets, in contrast, we do not find evidence for this negativity. Interestingly, the preferred model in sets 3, 5, and 7 is not ${\mathscr{M}}_{+}$, which allows for quantitative individual differences. Instead, it is ${\mathscr{M}}_{1}$, which specifies a common effect without individual differences. In sets 3 and 5, there is strong evidence for the common-effect model; the Bayes factors in favor of ${\mathscr{M}}_{1}$ are at least three orders of magnitude over the next competitor. In set 7, in contrast, the evidence is fairly ambiguous, indicating that the data do not contain sufficient resolution to adjudicate among the different models. In summary, whenever we find individual differences, they are qualitative in nature rather than quantitative.

We find strong evidence for these differences in five data sets. In at least two, however, we find evidence against them. How do these two data sets, sets 3 and 5, differ from the others? Looking for a psychological explanation, we note that in both sets the judgment phase was administered after a 1-week retention interval, whereas it took place within one experimental session in all other sets. If this difference was systematic, the influence of retention-interval length could tell us something about the nature of individual differences. It is possible that differences in cognitive performance (e.g., source recollection; Begg et al., 1992) rather than personality underlie qualitative differences in truth effects, and that these cognitive differences are affected for example by the length of the retention interval. Alternatively, however, there could be simple statistical reasons for this result: Data set 3 is rather small, thus allowing for strong influence of shrinkage and making it difficult to evidence true individual differences should they exist. Therefore, we should be careful not to overinterpret the results. Any post hoc explanation should be addressed and critically tested in future experiments.

## Classifying individuals

Who are these individuals that depreciate the validity of repeated statements? Posterior means, such as those in Figs. 1 and 2, do not provide enough information for classification because classification should depend on the underlying variability. A better approach to classifying individuals with truly negative truth effects is to assess the posterior probability that an individual’s estimate is less than 0.

**Fig. 5:**
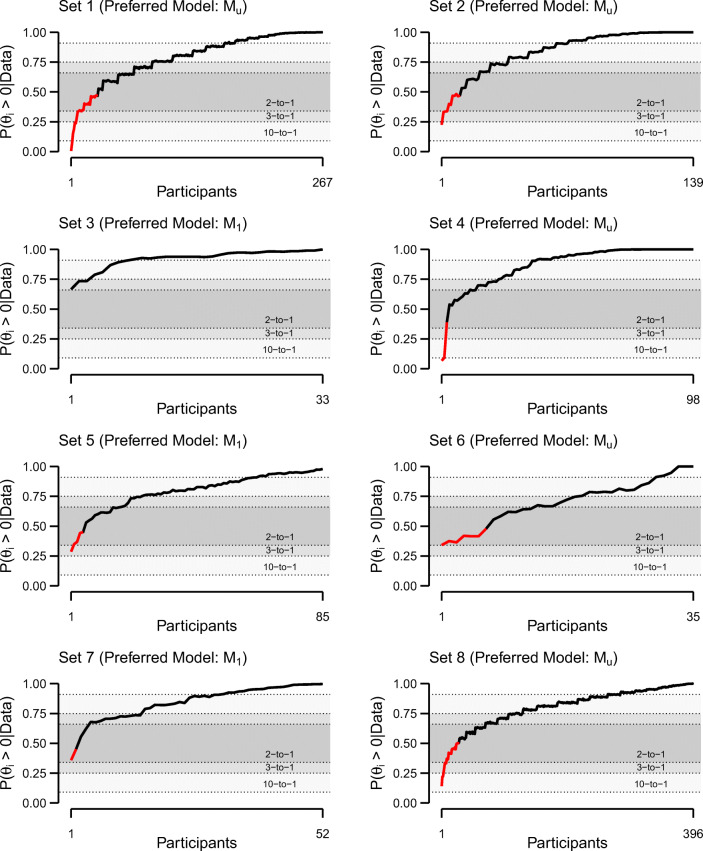
Individual posterior probabilities of a positive truth effect. Participants are ordered by observed effect size. The *red line* denotes individuals with negative posterior means (see Figs. 1 and 2)

Figure 5 shows the posterior probability of a *positive* truth effect for each individual, that is, *P*(*θ*_*i*_ > 0|Data). The red color denotes individuals with negative posterior means of *θ*_*i*_. To classify people, we may define a threshold denoting a desired level of certainty. If the posterior probability that *θ*_*i*_ is either positive or negative exceeds this threshold, we may classify the individual accordingly. Figure 5 contains three possible thresholds (denoted by the dotted lines) based on a probability of 10-to-1, 3-to-1, and 2-to-1. For the purposes of this article, we decided to classify individuals based on a probability of at least 3-to-1. Individuals with *P*(*θ*_*i*_ > 0|Data) ≥ .75 are classified as *positive truthers*. In contrast, individuals with *P*(*θ*_*i*_ > 0|Data) ≤ .25 are classified as *negative truthers*. The remaining participants with .25 < *P*(*θ*_*i*_ > 0|Data) < .75 are classified as undecided.

We acknowledge that the choice of a particular threshold is somewhat arbitrary. For our choice, in those data sets that showed strong evidence for qualitative individual differences, 69.30% of all participants are classified as positive truthers; only 1.28% are classified as negative truthers, leaving 29.41% as undecided. In set 6, we cannot define anyone as a negative truther with the desired level of certainty. This result shows that finding differences based on classification may be difficult even when a more global approach—model comparison—yields strong evidence that these differences exist.

A somewhat complimentary state-of-affairs occurs for sets 3 and 5. We found strong evidence for the absence of individual differences in these data sets. Yet, while no individuals classify as negative truthers, we still find 22.88% to be undecided. Thus, classification may find differences even when model comparison indicates that these differences are unwarranted.

The two scenarios illustrate the difficulty with classification: When we apply a classification approach, we may classify individuals as different even when there are no true individual differences. This state occurs because classification is local to the individual, and as such, it is more susceptible to noise than Bayes factor assessment of global patterns. Conversely, we may know from the Bayes factor global assessment that a set may have at least one individual with a true negative effect. Yet, based on individual posterior probabilities, it may be difficult to know which one that is. Note that this conflict between conclusions drawn from a classification approach and those from model comparison remains regardless of the particular classification threshold (see Fig. 5).

The aim of this paper is the global assessment of individual difference patterns in truth-effect experiments. If our overriding goal was to classify people, we could construct a latent-class classification model. In such a model, the normal in the unconstrained model could be replaced with a mixture of two states. One state would cover the positive truthers; and the distribution would be limited to positive true values. The other would cover the negative truthers, and the distribution would be limited to negative true values. If there is little mass toward zero, the model would have the effect of cleaving people clearly into two groups. A good example here is Houpt and Fifić (2017), who used this latent-class approach to classify people as using either serial or parallel processing in a systems factorial setting. The development of such a model is beyond the scope of this paper, but may prove useful in understanding the relationship between individual differences in the truth effect and other variables.

## Prevalence of qualitative differences

Instead of classifying individuals, we may take a more global perspective and ask how prevalent the negative truth effect is. To that end, we are no longer interested in who the negative truthers are, but rather how large the proportion of negative truthers in the population is. In the unconstrained model, we defined $\theta _{i} \sim \text {Normal}(\nu , \delta ^{2})$. Based on posterior estimates for *ν* and *δ*^2^, we can thus estimate the area of this distribution that is below 0. This area represents an estimate for the prevalence of qualitative differences, that is, the expected proportion of negative truthers in the population.

**Fig. 6:**
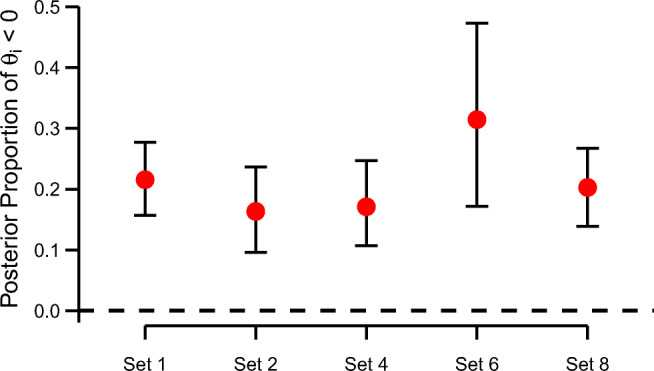
Posterior means and 95% credible intervals for the proportion of negative individual effects

A posterior estimate of this probability may be obtained using the MCMC outputs. For each posterior sample of *ν* and *δ*^2^, we obtain a posterior sample for the proportion of negative truthers. These samples converge to the appropriate posterior distribution, and the mean serves as a suitable estimate. Figure 6 shows the posterior mean and 95% credible interval for data sets 1, 2, 4, 6, and 8. The expected proportion is around .20 in all sets and the lower limit of all credible intervals is well above 0. This estimate is compatible with the model comparison results for these data sets, which yielded strong evidence for qualitative individual differences.

The analysis indicates that, given that there are qualitative individual differences, we can expect a substantial proportion of individuals to show a negative truth effect. This notwithstanding, we caution the reader not to overinterpret these results. One issue that is present is an undue dependency in this calculation on prior settings. The problem manifests on the prior of *g*_*θ*_. The Inverse-*χ*^2^ distribution has no mass on small values (see Fig. 4, lower tail), and as a result, values of *θ*_*i*_ may spread to a larger degree than is compatible with the data. We find that while the probability estimates are robust to different values of the prior scale $r^{2}_{\theta }$ for data sets with demonstrable individual differences (data sets 1, 2, 4, 6, and 8; see Fig. 6), they are unduly dependent on $r^{2}_{\theta }$ when there is a lack of resolution to detect such differences (data sets 3, 5, and 7).

## Sensitivity to prior settings

The Bayesian analysis presented herein requires the analyst to set the prior scale *r*^2^ on the signal-to-noise ratio *g*. The dependence of Bayesian analysis on prior settings is frequently criticized as posing a threat as it provides for uncounted researcher degrees of freedom (Simmons et al., 2011). Indeed, it seems reasonable to require that for the same data set, different researchers should reach the same conclusions. Yet, almost all Bayesians note that priors have effects on inference. To align Bayesian inference with the above desideratum, many Bayesian analysts actively seek to minimize the effects of prior settings (e.g.,Aitkin, 1991, Gelman et al., 2004, Kruschke, 2013, Spiegelhalter et al., 2002).

We do not subscribe to the view that minimization of prior effects is necessary or even laudable. In fact, all reasonable statistical procedures that we are aware of require the researcher to make decisions that will affect the inference (e.g., choosing the sample size). The choice of prior settings is important because it affects the predictions that models make about data. Therefore, these settings that affect the predictive accuracy of a model *should* affect our opinions about it in light of data.

Thus, when different researchers use different priors, they may reach different opinions about the data. Rouder et al., (2016) argue that so long as various prior settings are *justifiable,* the variation in results should be embraced as the legitimate diversity of opinion. When reasonable prior settings result in conflicting conclusions, we may infer that the data do not afford the precision to adjudicate among competing positions.

With this argument in mind, we may assess whether reasonable variation in prior settings affects Bayes factor conclusions about the nature of individual differences in the truth effect for the current data. To that end, we repeated the above analysis with a number of different prior settings. The critical settings are *r*_*ν*_ and *r*_*θ*_, which code the scale of effects. In the original analysis, we set *r*_*ν*_ and *r*_*θ*_ to 0.50 in value, meaning that we expected the variation in *ν* and *θ* to be about half the variation in repeated observations. Here, we allow each of these settings to be this value, half this value, and twice this value; and the factorial combination yields nine possible settings (see Table 3). We computed the Bayes factors for all models for all nine settings for all data sets to understand how reasonable variation in prior settings affects inference.

**Table 3:** Prior settings for sensitivity analysis

	Prior setting								
Parameter	A	B	C	D	E	F	G	H	*
*r*_*ν*_	1/4	1/4	1/4	1/2	1/2	1	1	1	1/2
*r*_*θ*_	1/4	1/2	1	1/4	1	1/4	1/2	1	1/2

*Note.* The asterisk codes the prior setting used in the previous analysis.

**Fig. 7:**
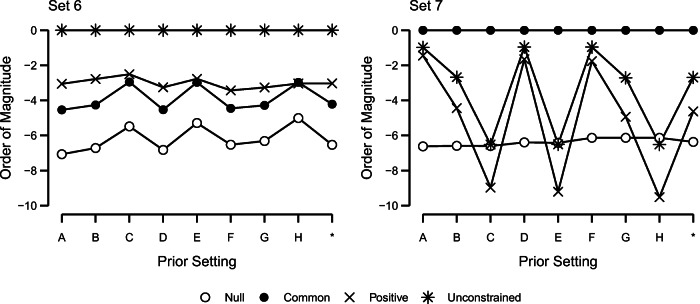
Sensitivity of Bayes factor model comparison to different prior settings. Shown are Bayes factors for each model against the preferred model. The *asterisk* denotes the prior setting used in the previous analysis. Details for each prior setting are shown in Table 3

For seven of eight data sets, model comparison was unaffected by reasonable variation in prior settings. As an illustration, the results for two data sets are depicted in Fig. 7. The figure shows the Bayes factors for all models relative to the preferred one in the previous analysis. On the right is data set 7, the most concerning. Here, the common-effect model is preferred only by a negligible amount depending on the prior setting, indicating a lack of a clear verdict between the models. This lack of resolution holds only for this data set. The left panel shows the case for data set 6, and we chose this set because, outside of data set 7, Bayes factors were most dependent on prior settings. Even so, the unconstrained model is preferred over all other models by at least a factor of 100 across the range of reasonable prior settings. In the remaining data sets (not shown), there is even more stability of Bayes factors across the ranges. Hence, across reasonable variation in prior settings, data sets 1, 2, 4, 6, and 8 show strong evidence for qualitative individual differences. In a similar fashion, the common-effect model is clearly preferred for all prior settings in data sets 3 and 5. Overall, the results presented here are robust to a wide range of reasonable prior opinion.

## General discussion

In this paper, we show a surprising finding. Although the truth effect is reliably obtained across many data sets, the effect itself is inconsistent across people. We are confident that in most experiments some people truly judge repeated statements as more valid than novel ones, while others truly judge them as less so. This effect is not just noise—the models indicate that this inconsistency occurs above and beyond trial-by-trial variation. What makes the finding surprising to us is that the result is in contrast to previous work with these individual-difference models. The modal result is that “everybody does”, that is, there are no qualitative individual differences in common cognitive effects such as Stroop and Flanker effects (Haaf and Rouder, 2017, 2019). In the repetition-induced truth effect, these differences exist, and they occur consistently across several data sets.

Does the presence of qualitative individual differences inform current cognitive theories of the truth effect? We think it should. A number of theoretical explanations have been proposed for the repetition-induced truth effect, for example, the recognition account (Bacon, 1979), the source-dissociation hypothesis (Arkes et al., 1991), the familiarity account (Begg et al., 1992), processing fluency (Reber & Schwarz, 1999), or the referential theory (Unkelbach & Rom, 2017). These accounts assume different underlying cognitive mechanisms, yet, they all make the same core prediction: repetition increases perceived validity. Unkelbach et al., (2019) summarize thusly: “No matter which mental processes may underlie the repetition-induced truth effect, on a functional level, repetition increases subjective truth” (p. 5). We argue, based on our analysis, that this statement is too general. In fact, we show what Davis-Stober and Regenwetter (2019) call the *paradox of converging evidence*: Across data sets, we find converging evidence that the statement holds on the mean level—yet, at the same time, we accumulate strong evidence that it doesn’t hold for everybody. Consequently, our results present converging evidence against theoretical positions that do not account for negative truthers.

This paper constitutes a first step by providing an answer to the fundamental question *if* there are qualitative individual differences in the truth effect. Having established such differences, the next step is to understand *why* they occur. One salient finding in this domain is that the overall truth effect can be reversed, that is, made negative, by certain experimental manipulations. Unkelbach and colleagues started with the proposition that easy-to-process statements are naturally more likely to be true (Unkelbach, 2007; Unkelbach & Stahl, 2009; see also Reber & Unkelbach, 2010; Unkelbach, 2006). In a set of creative experiments, these researchers reversed the correlation between fluency and truth, making difficult-to-read statements more likely to be true. With this correlation reversed, they observed a negative truth effect, that is, repeated statements, which are easier to process than novel statements, were now judged more likely to be false (but see Silva et al.,, 2016). One wonders if some participants have learned in their natural environment that ease-of-processing correlates with falseness, thus resulting in the observed qualitative individual differences.

Likewise, differences in memory ability might account for some of the individual differences patterns. We are most intrigued by the finding that there was evidence against individual differences in data sets where the interval between exposure and judgment lasted several days. Why would individual differences be attenuated or absent with increasing retention intervals? We suspect such a finding reflects an explicit memory-based effect (i.e., source recollection or memory for presented statements). As overall memory performance declines with increasing delay between exposure and judgment phase, these differences may diminish and, correspondingly, individual differences in the truth effect may disappear.

These post hoc explanations presented above are of course speculative. They form hypotheses to be addressed in future research. Based on our results, a promising way to examine the underlying mechanisms and possible covariates of individual differences in the truth effect is with a latent-class approach. Unlike correlational approaches, it relies on *ordinal* (i.e., In which direction is the effect?) rather than *metric* (i.e., How large is the effect?) measures. Given the strong evidence for qualitative individual differences in the majority of data sets, questions about who differs, when they differ, and why they differ are suitable to test and inform theories of the repetition-induced truth effect.

## Appendix A: Description of data sets

### Set 1

Set 1 contains data from an unpublished study by Nadarevic et al., (2012) on the association of personality traits and the truth effect. In this web-based study, participants were presented with 20 trivia statements, half of which were true, and half were false. Participants were informed that statements could be either true or false, and asked to assign each to one of five knowledge categories. In a subsequent phase, participants completed a number of personality questionnaires. Finally, participants were again presented with 20 trivia statements and asked to provide a truth rating on a scale from 1 to 5 for each statement. Ten of these statements had been presented in phase 1, the other ten statements were new. In total, 267 participants completed the study. Mean truth ratings for repeated statements were higher than for new statements, *M*_rep_ − *M*_new_ = 0.20 (*SD* = 0.34). A two-sided *t* test for paired samples revealed a significant truth effect, *t*(266) = 9.47, *p* < .001, Cohen’s *d* = 0.58.

### Set 2

Data set 2 is from Nadarevic and Rinnewitz (2011). In the exposure phase, participants were asked to assign 20 unknown trivia statements to one of five knowledge categories. After completing a personality questionnaire in the retention phase, participants were asked to provide truth ratings for 20 statements on a scale from 1 to 5. Ten of these statements were repeated from the exposure phase, the others were new. As an experimental manipulation, one group of participants was instructed to respond intuitively, while the other was asked to think carefully about each truth judgment. This manipulation did not have any systematic effect on truth judgments, however. Therefore, we did not include the factor condition in the analysis. The total sample comprised 139 participants. On average, truth judgments for repeated statements were higher than for new statements, *M*_rep_ − *M*_new_ = 0.28 (*SD* = 0.36). This difference is statistically significant, *t*(138) = 9.16, *p* < .001, Cohen’s *d* = 0.78.

### Set 3

Data set 3 is based on Experiment 1 in Nadarevic and Aßfalg (2017) who investigated the influence of warnings on the truth effect. For the analysis, we only included data from the control group, which received standard instructions and no warnings. The sample comprises 33 students who participated in the lab-based study. In phase 1, participants assigned 98 statements (including ten buffer statements) to knowledge categories. After a 1-week retention interval, they returned to the lab and provided truth statements on a scale from 1 to 6 for 44 statements from phase 1 and 44 new statements. Truth statements for repeated items were significantly higher than for new statements, *M*_rep_ − *M*_new_ = 0.11 (*SD* = 0.10), *t*(32) = 6.54, *p* < .001, Cohen’s *d* = 1.14.

### Set 4

For set 4, we used data from Nadarevic and Aßfalg’s (Nadarevic & Aßfalg, 2017) Experiment 2. It was identical to the first experiment with the exception that the exposure phase was directly followed by the judgment phase and participants rated 80 statements, half of which were repeated. As in the previous data set, we only included the control condition in the analysis. The set contains 98 participants. On the mean level, there is a significant truth effect: *M*_rep_ − *M*_new_ = 0.20 (*SD* = 0.23), *t*(97) = 8.61, *p* < .001, Cohen’s *d* = 0.87.

### Set 5

Data set 5 is based on Experiment 1 reported in Nadarevic and Erdfelder (2014). The authors investigated the influence of phase 1 task and retention interval between exposure and judgment phase on the truth effect. Unlike in the previous experiments, participants were asked to rate the statements’ validity already in phase 1. In two subsequent truth-judgment phases, participants rated the statements again: One was administered after 10 min (phase 2), another one after 1 week (phase 3). Half of the statements from phase 1 were repeated in phase 2, the other half was repeated in phase 3. Truth judgments in phase 1 and a retention interval of several days is a commonly used setting in truth-effect experiments (e.g., Hasher et al., 1977). In contrast, rating the statements’ truth in phase 1 and again after a short retention interval does not lead to a truth effect. Therefore, we only included truth judgments from phase 3 in the analysis. The sample comprised 85 participants and 88 truth judgments on a scale from 1 to 6 per participant. Analysis of mean truth ratings for repeated and new statements revealed a significant truth effect: *M*_rep_ − *M*_new_ = 0.05 (*SD* = 0.10), *t*(84) = 5.09, *p* < .001, Cohen’s *d* = 0.55.

### Set 6

Set 6 contains data from Experiment 2 in Nadarevic and Erdfelder (2014). Participants were assigned to one of two experimental conditions. In one condition, as in Experiment 1, participants rated the truth of each statement in phase 1. In the other condition, participants assigned the statements to knowledge categories. After a 10-min retention interval, participants in both groups provided truth statements for 88 statements, half of which were repeated from phase 1. We only included data from the category-rating condition in the analysis. The sample comprised 35 participants. Average truth ratings for repeated statements were significantly higher than for new statements, *M*_rep_ − *M*_new_ = 0.07 (*SD* = 0.17), *t*(34) = 2.50, *p* = .017, Cohen’s *d* = 0.42.

### Set 7

Data set 7 is based on Experiment 1 reported in Brashier et al., (2020). Similar to Nadarevic and Erdfelder (2014) Experiment 2, participants initially rated 60 statements either for truthfulness or for interest on a scale from 1 to 6. In the judgment phase, they provided truth ratings for these statements and 60 additional statements. We included the data from the interest-rating condition in the analysis. The data set contains 52 participants. The average difference in truth ratings was *M*_rep_ − *M*_new_ = 0.09 (*SD* = 0.12), revealing a significant truth effect: *t*(51) = 5.52, *p* < .001, Cohen’s *d* = 0.77.

### Set 8

For the last data set, we included data from Experiment 1 reported in Pennycook et al., (2018). Participants rated the interestingness of 14 trivia statements in the first phase. After a short retention interval, participants rated the same statements and 14 additional statements in terms of truthfulness on a scale from 1 to 6. Eight of these 28 statements were likely to be known by all participants, the other 20 statements were largely unknown. In the reanalysis, we included truth ratings for the 20 unknown statements. We excluded 13 participants due to missings that occurred during the judgment phase. The final data set comprises 396 participants. On average, truth judgments for repeated statements were higher than for new statements, *M*_rep_ − *M*_new_ = 0.14 (*SD* = 0.27), revealing a significant truth effect: *t*(395) = 10.08, *p* < .001, Cohen’s *d* = 0.51.

## Appendix B: Prior specifications

The parameters *α*_*i*_, *β*, *μ*, and *σ*^2^ are common to all models. We place a noninformative and scale-invariant prior called *Jeffreys prior* (Jeffreys, 1961) on the non-effect parameters *μ* and *σ*^2^:

7 $$ \pi(\mu,\ \sigma^{2}) \propto \frac{1}{\sigma^{2}}. $$

Priors on the remaining parameters are defined as functions of *σ*^2^, the variance of the observed variable. We specify the following prior for person-specific intercepts

8 $$ \begin{array}{ll} \alpha_{i} &\overset{\text{iid}}{\sim} \text{Normal}(0,\ g_{\alpha} \sigma^{2}),\\ g_{\alpha} &\sim \text{Inverse-}\chi^{2}(r_{\alpha}^{2}). \end{array} $$

We expected the variability in people’s individual baselines to be about twice as large as the inter-trial variability. Therefore, we set $r_{\alpha }^{2} = 2^{2}$. Note that the inverse-*χ*^2^ distribution has a heavy tail and, given sufficient data, will be overruled if the observed variability is larger than expected.

We apply a similar reasoning to the effect of factual truth, *β*. We define

9 $$ \begin{array}{ll} \beta &\sim \text{Normal}(0,\ g_{\beta} \sigma^{2}),\\ g_{\beta} &\sim \text{Inverse-}\chi^{2}(r_{\beta}^{2}), \end{array} $$

and set $r_{\beta }^{2} = 2^{2}$. Sensitivity analyses revealed that results did not differ meaningfully for any choice of $r_{\alpha }^{2},\ r_{\beta }^{2} \in [0.25, 16]$.

## Footnotes

1 Set 1: https://osf.io/6wv4z/; Set 2: https://osf.io/3uaj7/; Sets 3 & 4: https://osf.io/5pfa2/; Sets 5 & 6: https://osf.io/eut35/; Set 7: https://osf.io/b4szp/; Set 8: https://osf.io/txf46/

2 Note that some model parameters are labeled equivalently across models. This is to indicate that these parameters carry the same substantive interpretation (e.g., *δ*^2^ represents individual variability both in the context of ${\mathscr{M}}_{U}$ and ${\mathscr{M}}_{+}$).
